# Causal role of immune cells in psoriasis: a Mendelian randomization analysis

**DOI:** 10.3389/fimmu.2024.1326717

**Published:** 2024-03-15

**Authors:** Anning Wang, Jingyuan Zhang

**Affiliations:** ^1^ Dalian Dermatosis Hospital, Dalian, Liaoning, China; ^2^ Department of Traditional Chinese Medicine, First Affiliated Hospital of Dalian Medical University, Dalian, Liaoning, China

**Keywords:** Mendelian randomization, psoriasis, immune cells, T cell, B cell

## Abstract

**Background:**

A growing body of evidence has shown that immune cells are linked to psoriasis. It is, however, still unclear if these associations reflect a relationship of cause and effect.

**Objective:**

We employed a two-sample Mendelian randomization (MR)-based study to elucidate the probable causative connection between immune cells and psoriasis.

**Methods:**

Summary information for psoriasis (Ncase = 5,427, Ncontrol = 479,171) was obtained from the European Bioinformatics Institute. Summarized statistical information on 731 immune cell features, including morphological parameters (MP; n = 32), relative cell number (n = 192), median fluorescence intensity (MFI) of surface antigens (n = 389), and absolute cell number (n = 118), was obtained from the genome-wide association studies (GWAS) catalog. The research consisted of forward MR analysis, in which immune cell traits were used as the exposure factor, and psoriasis was the outcome, as well as reverse MR analysis, in which psoriasis was used as the exposure factor, and immune cell traits were the outcome. We ran numerous sensitivity analyses to ascertain the study results for robustness, heterogeneity, and potential multiple-biological effects.

**Result:**

This research determined a probable causative connection between immune cells and psoriasis. In particular, we identified 36 distinct types of immune cells that are potentially causally linked to psoriasis.

**Conclusion:**

Our findings indicate strong causal correlations between 36 immunological phenotypes and psoriasis, thus, directing future clinical trials.

## Introduction

With an estimated incidence of 2%–3% globally, psoriasis is a chronic inflammatory skin disease defined by genetics and mediated by the innate or adapted immunological system (1). Psoriasis is typified by red plaques coated in silvery-white scales, punctate hemorrhages, and inflammatory infiltrates. Approximately 30% of patients experience arthritic symptoms, and approximately 50% experience aberrant nail alterations (1). People with psoriasis are more likely to have psychological wellbeing problems because of the long-term nature of their illness and recurring nature of the condition. They may also experience social obstacles, psychological stress, and unpleasant feelings that interfere with their day-to-day activities and professional endeavors (2, 3). Topical drugs (keratolytics, steroids, retinoids, etc.), oral drugs (immunosuppressants, retinoids, biologics, etc.), phototherapy (ultraviolet B irradiation, etc.), and biologics are among the therapies for psoriasis. Biologics can target particular immune molecules to lessen inflammatory responses and symptoms, whereas oral medicines and physiotherapy have some adverse effects (2, 3).

In 2019, psoriasis accounted for 6.84% of all new instances of immune-mediated inflammatory diseases, which are increasingly widespread globally (4). The epithelial immune microenvironment of epithelial tissue is composed of five prevalent elements as follows: immune cells, microbiota, barriers, epithelial cells, and peripheral nerve endings, with immune cells being the most dominant (5). Atypical keratinocyte differentiation and immune cell activation are the causes of psoriasis (6). As an example, the emergence of psoriasis disease is stimulated by the heightened expression of T helper 17 (Th17) cells and the secretion of interleukin (IL)-17A (1). Immune mediators of the IL-17 pathway have been found to produce abnormalities in epidermal keratinocyte proliferation and differentiation, which is the cause of psoriasis. Treatments that target IL-17 and IL-17A have been licensed for use in the clinic and have shown great success (7). However, not every psoriasis patient has a good clinical outcome through the application of IL-17 pathway immunological agents; thus, we still need to find more immune cells affecting psoriasis to solve the adverse effects of psoriasis more thoroughly. Furthermore, because of small sample numbers, poor study designs, and confounding variables outside the purview of current research, the conclusions drawn thus far about the connection between immunological inflammation and psoriasis are incomplete.

Mendelian randomization (MR) serves as an analytical method based on genetic data, which is primarily used in epidemiology to ascertain disease causes. MR research aims to determine whether individuals with specific genetic differences are prone to acquire diseases than people without them by “randomizing” their genes based on one or more alleles influencing risk factors (8). By employing genetic diversity as a key factor in two-sample MR, we were able to assess how immune cell characteristics affect the emergence of psoriasis and eliminate other potential influencing factors. Altering the characteristics of immune cells could be beneficial in preventing or treating psoriasis, provided that the research findings establish a causative connection.

## Materials and methods

### Study design

**Figure 1:**
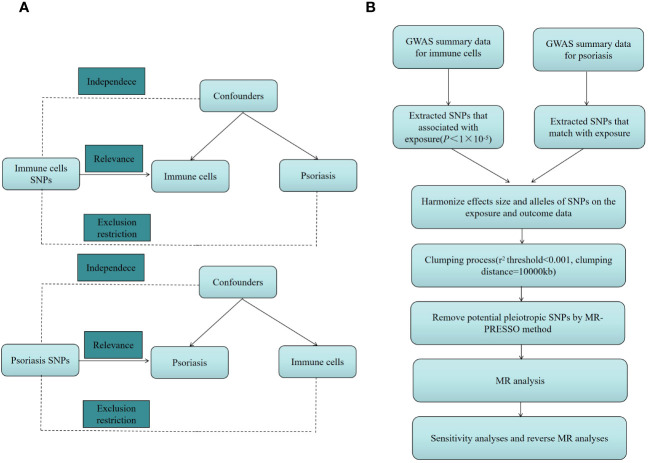
**(A)** The diagram of MR assumption. **(B)** The diagram of MR analysis processing. SNPs, single nucleotide polymorphisms; GWAS, genome-wide association study; MR, Mendelian randomization; MR-PRESSO, Mendelian Randomization Pleiotropy RESidual Sum and Outlier (MR-PRESSO) test.

We investigated the causal link between 731 immune cell characteristics and psoriasis using two-sample MR techniques. MR, a statistical approach, employs genetic variants as instrumental variables (IVs) for accessing causation. IVs must meet three essential presumptions to guarantee the validity of causal inferences: (1) Genetic variation and exposure are intimately correlated, meaning that genetic variation can influence immune cell characteristics, potentially impacting psoriasis. (2) Confounders are additional variables that may impact both exposure and outcome; genetic variants were not associated with any potentially confounding factors among exposures and outcomes. Genetic variation cannot be used as a legitimate IV if it is linked to these confounding factors. (3) The only method by which genetic variation may influence psoriasis is by altering immune cell properties; genetic variation cannot influence results by any mechanism besides exposure. An outline of the research design is depicted in **Figure 1**.

### Data source for the GWAS on psoriasis

Aggregated genome-wide association studies (GWAS) statistics for psoriasis are from the European Bioinformatics Institute. A genomic association study was conducted on 484,598 European individuals (N case = 5,427, N control = 479,171), and 9,587,836 single nucleotide species numbers were analyzed after quality control and estimation (SNP; https://gwas.mrcieu.ac.uk/datasets/ebi-a-GCST90038681/).

### Data source for the GWAS on immune cells

This research aimed to investigate the causal relationship between psoriasis and 731 immunological traits. The GWAS that yielded extensive statistical summaries of immunological features (accession numbers GCST0001391 to GCST0002121) was the source of the data (9). The 731 distinct immunophenotypes in the dataset are categorized into four groups as follows: morphological parameters (MP; n = 32), relative cell counts (n = 192), surface antigen median fluorescence intensity (MFI; n = 389), and absolute cell counts (n = 118). The collection also includes information on several cell types, such as natural killer cells, monocytes, B cells, granulocytes, T cells, regulatory T cells (Tregs), and dendritic cells (CDCs).

### Selection of IVs

Recent research indicates that the significance level for each immune trait in IVs was established at P<1 × 10^−5^ (10, 11). Then the threshold of *r* ^2^ was set to 0.001 and the kilobase pair (kb) to 10,000 to aggregate SNPs. When *r* ^2^ > 0.001 and kb < 10,000, SNPs are removed from the current analysis, ensuring that there is no linkage disequilibrium (LD) correlation (12). The heterogeneity test excluded significant heterogeneous SNPs with an F statistic <10 and retained effective SNPs as an instrumental variable (13).

### Statistical analysis

All analyses were carried out using the R 4.3.1 program.

MR research with two samples and sensitivity analysis: two-sample MR studies of many immune cell-related characteristics were conducted in our work. For these analyses, we employed the inverse variance weighted (IVW), MR-Egger, and weighted median (WM) approaches. Using the IVW approach, the primary result was determined, maintaining the statistical analysis level of importance at p < 0.05. By making the influence of variation on the outcome inversely proportional to its variance, IVW assigns greater weight to variants with lower variance resulting in a more accurate estimate. By utilizing the inverse of the outcome’s variance to weight the more dependable, lower variance estimates, the approach eliminates the intercept from the regression fit (14). We used MR-Egger regression for pleiotropy evaluation, which is the process by which one gene influences several traits. A notable intercept (p < 0.05) indicates considerable horizontal pleiotropy, and this approach incorporates an intercept evaluation to determine pleiotropy (15). Using Cochran’s Q statistic, which gauges how consistently genetic variation affects phenotypes, we also looked at heterogeneity. Significant variability in the impact of several genetic variations is shown by a substantial Cochran’s Q (p < 0.05) (16). The WM approach was used for heterogeneous data that did not exhibit pleiotropic effects (p > 0.05) (15).

MR analysis done in reverse: We examined the possibility of reverse causality and looked into whether immune cell characteristics are impacted by psoriasis using the same MR methodology. Psoriasis was regarded as the exposure factor in this reverse MR study, and different immune cell features were deemed the results.

## Results

### Investigating the influence of immune cells on psoriasis

**Figure 2:**
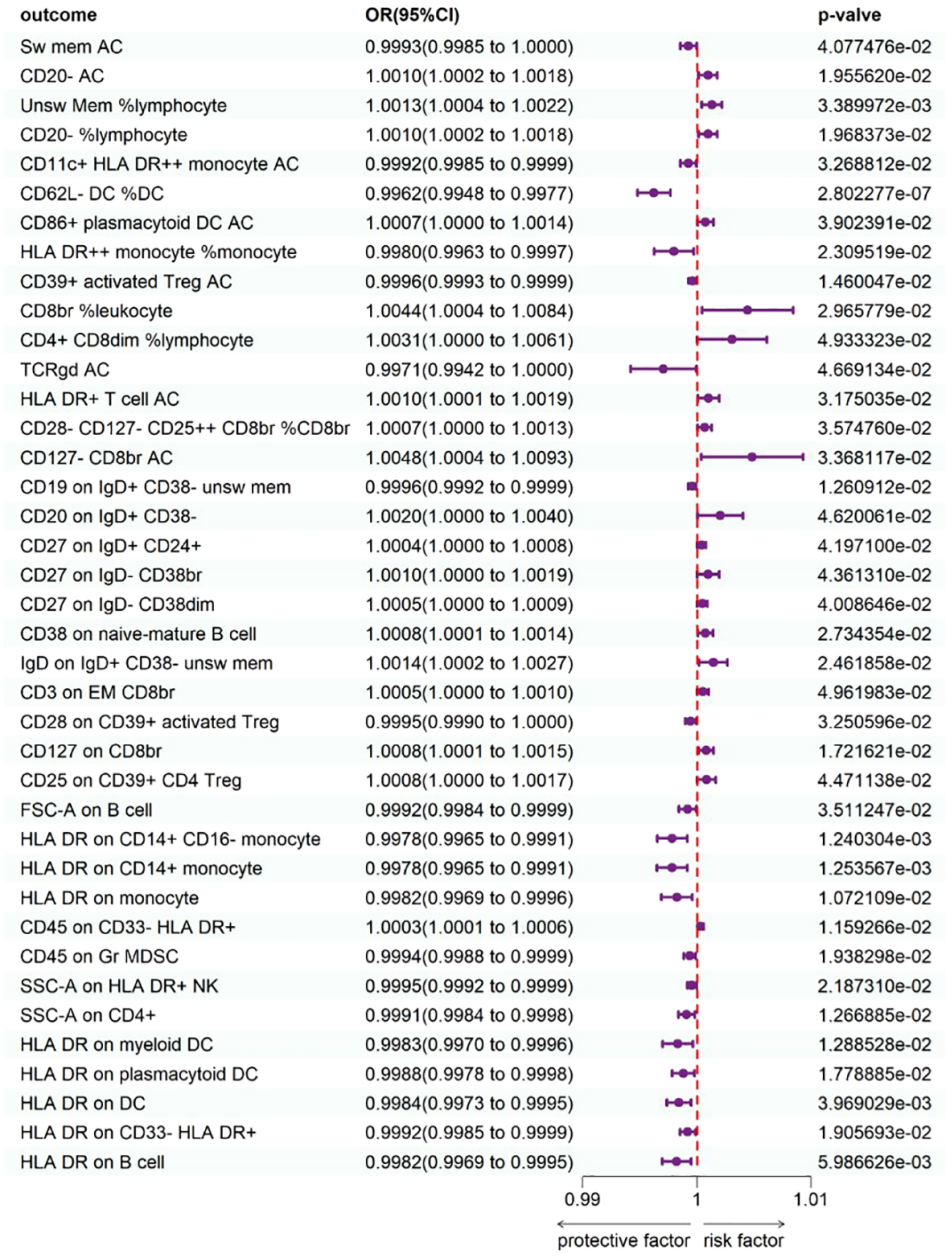
Forest plot shows the expression causality of immune cells for psoriasis.

**Figure 3:**
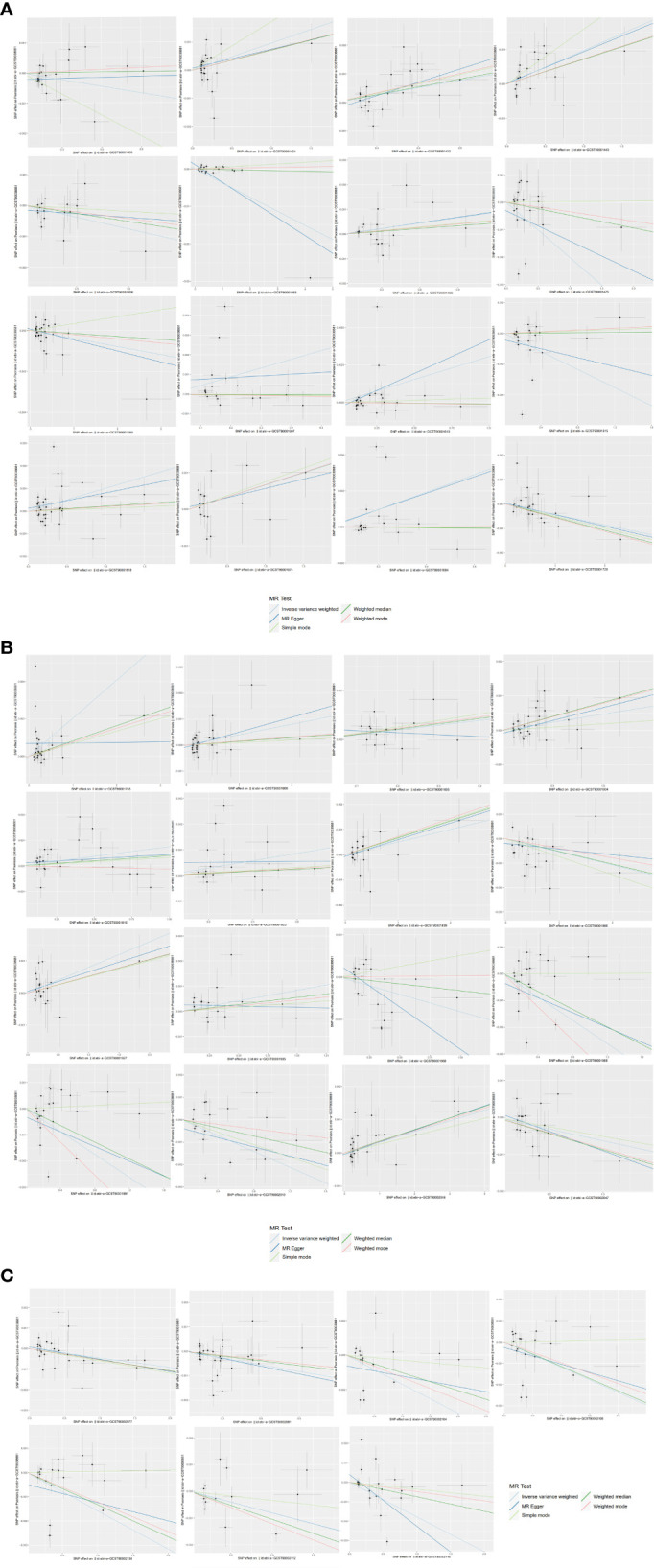
**(A–C)** Scatter plots of the above results indicate stability.

**Figure 4:**
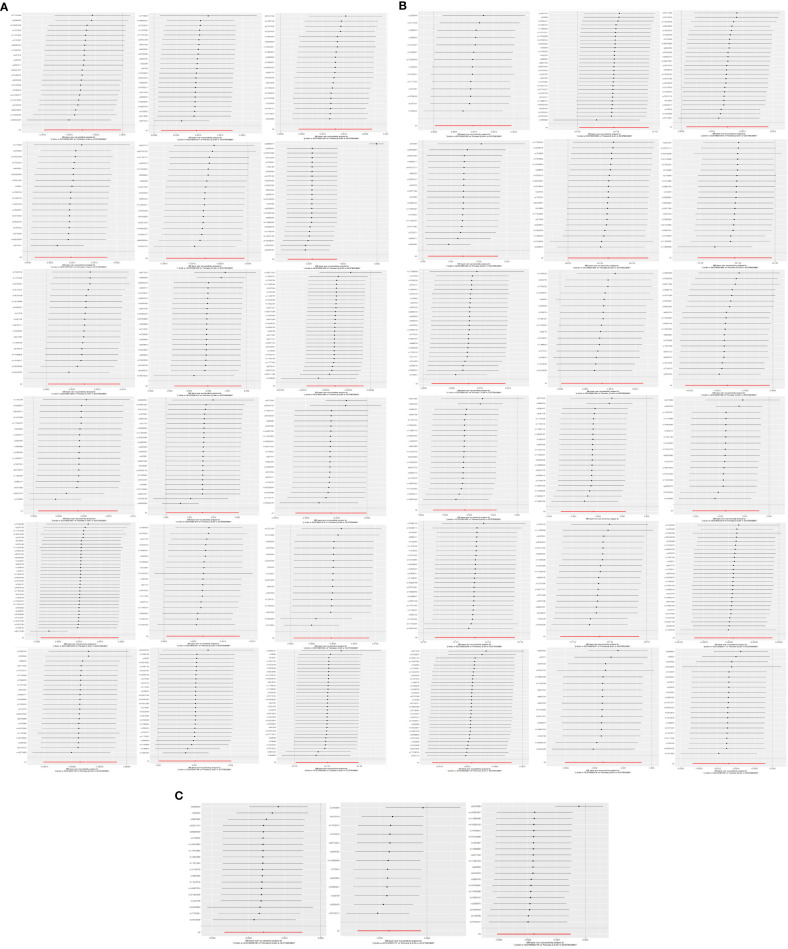
**(A–C)** The “leave-one-out” method of the above results indicates stability.

To study the causal influence of immune cell types on psoriasis, a two-sample MR analysis was performed, utilizing the IVW technique as a major analytical strategy and removing potential confounders and horizontal pleiotropy. A total of 39 immunophenotypes linked with psoriasis were discovered (p < 1 × 10^−5^) (**Figure 2**). Scatter plots and the “leave-one-out” approach proved the results’ stability. **Figures 3A–C** show the scatter plots, and **Figures 4A–C** show the “leave-one-out” analyses.

Within B cell panels, nine traits were associated with a heightened risk of psoriasis: CD20− AC [IVW: odds ratio (OR) 1.0010, 95% confidence interval (CI) 1.002–1.0018; p = 1.955620e−02], unsw mem %lymphocyte (IVW: OR 1.0013, 95% CI 1.0004–1.0022; p = 3.389972e−03), CD20− %lymphocyte (IVW: OR 1.0010, 95% CI 1.0002–1.0018; p = 1.968373e−02), CD20 on IgD+ CD38− (IVW: OR 1.0020, 95% CI 1.0000–1.0040; p = 4.620061e−02), CD27 on IgD+ CD24+ (IVW: OR 1.0004, 95% CI 1.0000–1.0008; p = 4.197100e−02), CD27 on IgD− CD38br (IVW: OR 1.0010, 95% CI 1.0000–1.0019; p = 4.361310e−02), CD27 on IgD− CD38dim (IVW: OR 1.0005, 95% CI 1.0000–1.0009; p = 4.008646e−02), CD38 on naive-mature B cell (IVW: OR 1.0008, 95% CI 1.0001–1.0014; p = 2.734354e−02), IgD on IgD+ CD38− unsw mem (IVW: OR 1.0014, 95% CI 1.0002–1.0027; p = 2.461858e−02). Conversely, two traits showed protective connections with psoriasis: sw mem AC (IVW: OR 0.9993, 95% CI 0.9985–1.0000; p = 4.077476e−02), CD19 on IgD+ CD38− unsw mem (IVW: OR 0.9996, 95% CI 0.9992–0.9999; p = 1.260912e−02).

Within TBNK panels, three traits were associated with a heightened risk of psoriasis: CD8br %leukocyte (IVW: OR 1.0044, 95% CI 1.0004–1.0084; p = 2.965779e−02), CD4+ CD8dim %lymphocyte (IVW: OR 1.0031, 95% CI 1.0000–1.0061; p = 4.933323e-02), and HLA-DR+ T-cell AC (IVW: OR 1.0010, 95% CI 1.0001–1.0019; p = 3.175035e-02). Conversely, seven traits showed protective connections with psoriasis: HLA-DR++ monocyte %monocyte (IVW: OR 0.9980, 95% CI 0.9963–0.9997; p = 2.309519e−02), CD39+ activated Treg AC (IVW: OR 0.9996, 95% CI 0.9993–0.9999; p = 1.460047−02), TCRgd AC (IVW: OR 0.9971, 95% CI 0.9942–1.0000; p = 4.669134e−02), FSC-A on B cell (IVW: OR 0.9992, 95% CI 0.9984–0.9999; p = 3.511247e−02), SSC-A on CD4+ (IVW: OR 0.9991, 95% CI 0.9984–0.9998; p = 1.266885e−02), HLA-DR on B cell (IVW: OR 0.9982, 95% CI 0.9969–0.9995; p = 5.986626e−03), SSC-A on HLA-DR+ NK (IVW: OR 0.9995, 95% CI 0.9992–0.9999; p = 2.187310e−02).

Within cDC panels, one trait was linked with increased psoriasis risk: CD86+ plasmacytoid DC AC (IVW: OR 1.0007, 95% CI 1.0000–1.0014; p = 3.902391e−02). Conversely, five traits showed protective connections with psoriasis: CD11c+ HLA-DR++ monocyte AC (IVW: OR 0.9992, 95% CI 0.9985–0.9999; p = 3.268812e−02), CD62L− DC %DC (IVW: OR 0.9962, 95% CI 0.9948–0.9977; p = 2.802277e−07), HLA-DR on myeloid DC (IVW: OR 0.9983, 95% CI 0.9970–0.9996; p = 1.288528e−02), HLA-DR on plasmacytoid DC (IVW: OR 0.9988, 95% CI 0.9978–0.9998; p = 1.778885e−02), HLA-DR on DC (IVW: OR 0.9984, 95% CI 0.9973−0.9995; p = 3.969029e−03).

Within the Treg panels, three traits were associated with a heightened risk of psoriasis: CD28−CD127−CD25++ CD8br %CD8br (IVW: OR 1.0007, 95% CI 1.0000–1.0013; p = 3.574760e−02), CD127−CD8br AC (IVW: OR 1.0048, 95% CI 1.0004–1.0093; p = 3.368117e−02), and CD127 on CD8br (IVW: OR 1.0008, 95% CI 1.0001–1.0015; p = 1.721621e−02). Conversely, two traits showed protective connections with psoriasis: CD28 on CD39+ activated Treg (IVW: OR 0.9995, 95% CI 0.9990–1.0000; p = 3.250596e−02), CD25 on CD39+ CD4 Treg (IVW: OR 0.9984, 95% CI 0.9973–0.9995; p = 3.969029e−03).

Surprisingly, all three significant traits within monocyte panels were protective: HLA-DR on CD14+ CD16− monocyte (IVW: OR 0.9978, 95% CI 0.9965–0.9991; p = 1.240304e−03), HLA-DR on CD14+ monocyte (IVW: OR 0.9978, 95% CI 0.9965–0.9991; p = 1.253567e−03), and HLA-DR on monocyte (IVW: OR 0.9982, 95% CI 0.9969–0.9996; p = 1.072109e−02).

Within Myeloid cell panels, one trait was linked with increased psoriasis risk: CD45 on CD33−HLA-DR+ (IVW: OR 1.0003, 95% CI 1.0001–1.0006; p = 1.159266e−02). Conversely, two traits showed protective connections with psoriasis: CD45 on Gr MDSC (IVW: OR 0.9994, 95% CI 0.9988–0.9999; p = 1.938298e−02), and HLA-DR on CD33−HLA-DR+ (IVW: OR 0.9992, 95% CI 0.9985–0.9999; p = 1.905693e−02).

One trait with Maturation stages of T-cell panels was linked with increased psoriasis risk: CD3 on EM CD8br (IVW: OR 1.0005, 95% CI 1.0000–1.0010; p = 4.961983e−02).

Our sensitivity analysis supported our findings. Heterogeneity and horizontal pleiotropy analyses verified the strength of our findings. The leave-one-out analysis supported these conclusions.

### Investigating the influence of psoriasis on immune cells

**Figure 5:**
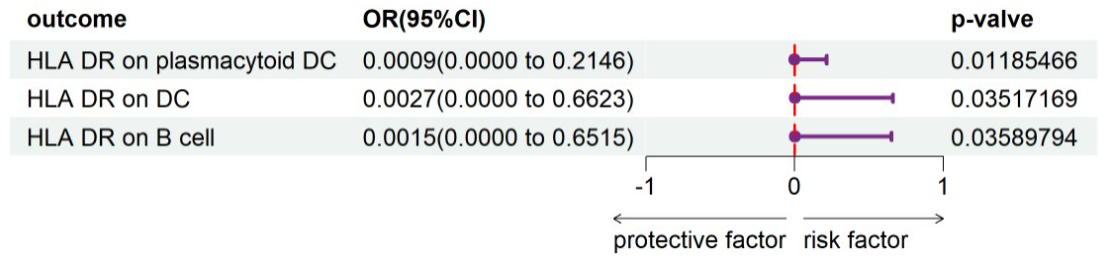
Forest plot shows the expression causality of psoriasis for immune cells.

**Figure 6:**
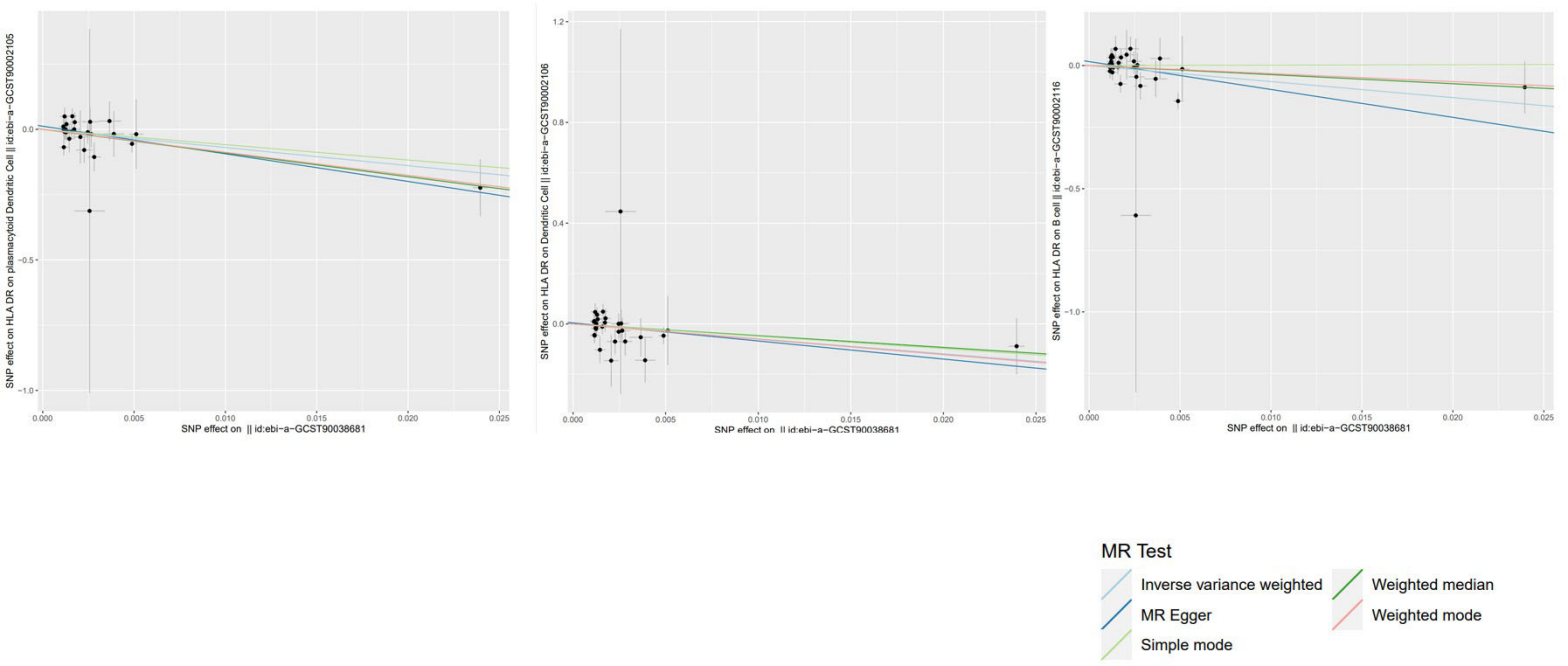
Scatter plots of the above results show the stability of the causal effect of psoriasis on immune cells.

**Figure 7:**
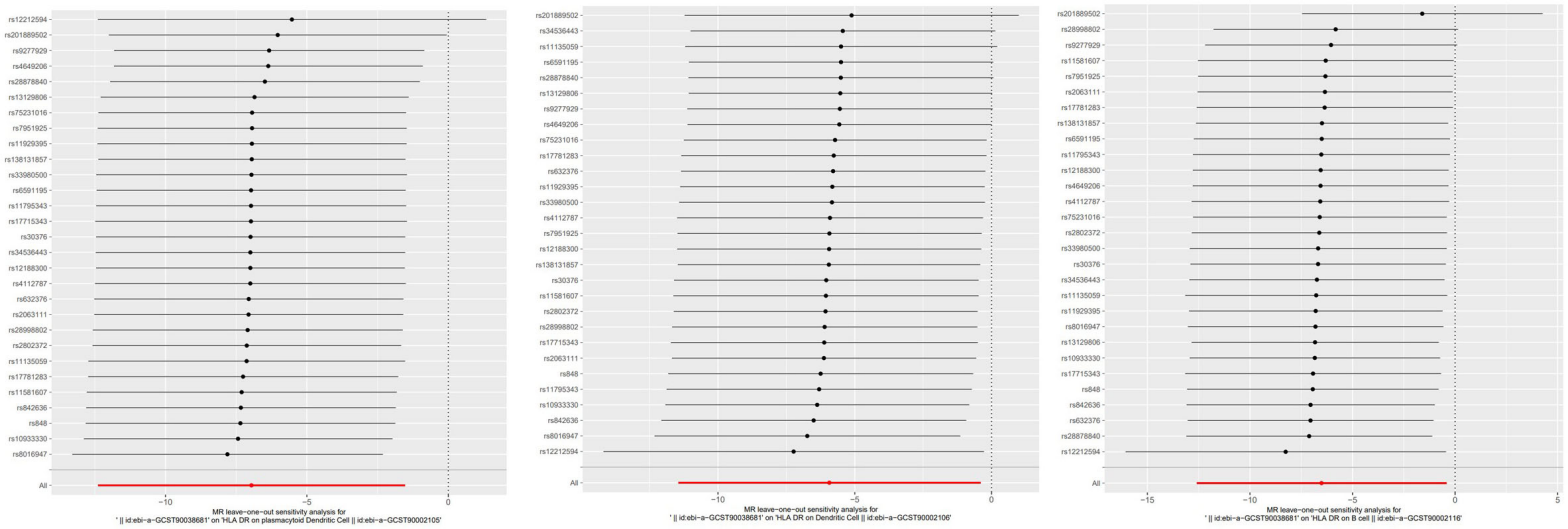
The “leave-one-out” method of the above results indicates the stability of the causal effect of psoriasis on immune cells.

To evaluate the reverse causal influence of psoriasis on the 39 positive immune cell features found, two-sample reverse MR analyses were carried out, primarily utilizing the IVW approach. When the genome-wide significance threshold was set at p < 5 × 10^−8^, psoriasis had no causal influence on immunological phenotypes. To discover additional SNPs linked with immune phenotypes, the genome-wide significance threshold was set at p < 1 × 10^−6^. Psoriasis was discovered to have an inverse causal association with HLA-DR on plasmacytoid DC, DC, and B cell. Within cDC panels: HLA-DR on plasmacytoid DC (IVW: OR 0.0009, 95% CI 0.0000–0.2146; p = 0.01185466), HLA-DR on DC (IVW: OR 0.0027, 95% CI 0.0000–0.6623; p = 0.03517169). One trait with TBNK panels is HLA-DR on B cell (IVW: OR 0.0015, 95% CI 0.0000–0.6515; p = 0.03589794). Psoriasis is related to higher levels of all three traits (**Figure 5**). Our sensitivity analysis supported our findings. Heterogeneity and horizontal pleiotropy analyses verified the strength of our findings. The leave-one-out analysis supported these conclusions. **Figure 6** shows the scatter plots, and **Figure 7** shows the “leave-one-out” analyses.

## Discussion

We used openly accessible genetic information to evaluate the link between 731 immune cell profiles and psoriasis. This is a novel MR investigation to examine the link between several immunophenotypes and psoriasis. Our investigation identified 36 immunological characteristics that had a causal influence on psoriasis. These findings shed fresh light on the pathophysiology of psoriasis and lay a theoretical foundation for future therapy.

Most research has concentrated on “single-cell RNA sequencing,” which investigates the transcriptome, or RNA sequence, of specific cells in tissues (17). Single-cell RNA sequencing demonstrated that Th17 and CD8 T cells all perform significant part in the onset and illness of psoriasis (17). To develop into Th17 cells, CD4+ cells are stimulated by T-cell receptor antigens and activated by cytokines released by antigen-presenting cells (17). Thus, this suggests that CD4 plays an equally important role in psoriasis. Our findings clearly show that CD4+ and CD8 T-cell panels can cause psoriasis.

Tregs serve a vital role in immunological homeostasis preventing autoimmunity by suppressing immune responses. Impairment of the adenosine signaling pathway in psoriasis may result in Treg suppression loss. CD39 transforms extracellular adenosine triphosphate to adenosine monophosphate, which is then inactivated via a variety of translational mechanisms, therefore, reducing immunosuppression (18). This enzymatic activity, in which CD39 is the main modifier, has an immunoregulatory function and plays a crucial role in immunomodulation through modifying the cell environment from a pro-inflammatory state driven by ATP to an anti-inflammatory state dominated by ADO (19). In this investigation, we found that CD28 on CD39+-activated Treg and CD25 on CD39+ CD4 Treg were protective against psoriasis. Mora-Velandia et al. showed that CD127 is expressed by traditional innate lymphoid cells, while non-classical CD127− populations have recently been identified. They also discovered a high expression level of the CD127− population in psoriasis patients (20). Our study reveals that CD28− CD127− CD25++ CD8br%CD8br, CD127− CD8br AC, and CD127 on CD8br may all enhance the chance of getting psoriasis.

Psoriasis is commonly associated with T cells, although the effect of B cells in its etiopathogenesis has received less attention. The presence of CD19+ B-cell subsets in psoriasis patients’ skin lesions has been associated with disease severity (21). Our data imply that certain B-cell panels, such as CD20−AC, unsw mem %lymphocyte, CD20− %lymphocyte, CD20 on IgD+ CD38−, CD27 on IgD+ CD24+, CD27 on IgD− CD38br, CD27 on IgD− CD38dim, CD38 on naive-mature B cell, and IgD on IgD+ CD38−unsw mem, are related to an elevated risk of psoriasis. Others, such as CD19 on IgD+ CD38−unsw mem and sw mem AC, provide protection. This highlights the fact that B cells are inseparable from the developmental regression of psoriasis. There is a need for more investigations into the complex involvement of B cell-associated groups in psoriasis rather than focusing only on T cell-associated immune cells.

HLA-DR is a particle of the human white blood cell antigen system that may have a role in immune response regulation and is a key immune response component (22). Research suggests that HLA-DR may be involved in the immune response to psoriasis and have a crucial role in the growth and advancement of psoriasis (23). Patients with psoriasis, in particular, have considerably elevated HLA-DR expression in their skin cells, which might be attributed to the immune system’s attack on the skin cells (22, 23). Ferenczi et al. emphasized that most T cells in the lesion sites of psoriasis patients are constantly active and that HLA-DR is substantially expressed in them (24). However, Yin et al. discovered that individuals with psoriasis exhibited a smaller proportion of HLA-DR expression than healthy controls (25). Interestingly, our results reflect this complexity. This study found that HLA-DR + T-cell AC contributes to the etiology of psoriasis. However, HLA-DR in DC cells, myeloid cells, monocytes, and other cell types protects against psoriasis. Our data clearly show that the majority of immune characteristics in DC cells and all immunological features in monocytes contribute to the treatment of psoriasis and are linked with HLA-DR. Remarkably, we found an inverse causal connection among HLA-DR and plasmacytoid DC, DC, and B cells, and psoriasis. Our results reveal a reduction in HLA-DR and plasmacytoid DC, DC, B cells linked with psoriasis, although psoriasis curiously increases these features implying an underlying negative feedback loop. The inflammatory response in psoriasis is multifaceted, and HLA-DR is likely to have an essential role to play in balancing the several factors that contribute to injury and the healing of the disease.

Furthermore, multiple experimental investigations have demonstrated that monocyte CD14 expression is much higher in psoriasis patients, which might be attributed to inflammatory mediators in psoriasis patients’ skin tissues driving monocyte CD14 expression. Zhang et al. discovered that CD14+ monocytes overexpressed BTN3A1 on their surface in psoriasis patients’ peripheral blood and skin lesions. This led to abnormal activation of Vγ9Vδ2 T cells, which secreted a large amount of IFN-γ, which upregulated BTN3A1 on monocytes, creating a vicious circle (26). However, unlike the previous investigations, the current study discovered that monocyte CD14 had a protective impact against psoriasis. This underlines the need for further investigation of the nuanced involvement of monocyte CD14-related phenotypes in psoriasis and the immunomodulatory modalities involved.

Our work uses the MR technique to discover probable linkages between immune cells and psoriasis risk going further traditional association studies. MR greatly reduces confounding by utilizing genetic variation as an instrumental variable, which is a critical strategy for overcoming frequent hurdles in observational investigations (27). This approach provides a detailed understanding of immune systems and, more importantly, assists in the discovery of immune traits that could raise or reduce the risk of psoriasis providing critical insights for the development of specific therapies. This research will be essential to the quick development of more precise and personalized psoriasis prevention and treatment strategies.

The study is an exploratory research and the most recent to give information on the general causal link between immune cells and psoriasis. Our objective was to investigate as many immunophenotypes as possible that may impact psoriasis episodes, therefore, setting the framework for future research. As a result, we did not apply multiple testing corrections. In reverse causality studies, relatively permissive criteria were utilized to evaluate the study outcomes, which may have raised the likelihood of false positives. The work relied on MR analysis with an ensemble of SNP data from the GWAS database. The aggregation of these data lacks the collection of individualized information, which may explain why some of the results of this study differ from those of previous trials analyzing individuals. Nonetheless, our dataset’s wide range of genetic diversity provides a good platform for investigating broader correlations between immune cells and psoriasis risk. Concisely, a comprehensive two-way MR analysis found an association between immune cells and psoriasis emphasizing the intricate relationships between immune cells and psoriasis. This opens up new paths for researchers to examine the role of the immune system in psoriasis and encourages more research into the immunology of psoriasis suggesting interesting possibilities for future immunotherapy methods.

## Conclusion

The current study discovered that immune cells play an important role in psoriasis and provides a valid analytical basis for future research on targeted immunomodulation in psoriasis patients. However, further clinical and basic research is required to support this concept.

## Data availability statement

The original contributions presented in the study are included in the article/supplementary material. Further inquiries can be directed to the corresponding author.

## Author contributions

AW: Writing – original draft, Writing – review & editing. JZ: Writing – review & editing.
